# Pleural ultrasonography versus chest radiography for the diagnosis of pneumothorax: review of the literature and meta-analysis

**DOI:** 10.1186/cc13016

**Published:** 2013-09-23

**Authors:** Saadah Alrajab, Asser M Youssef, Nuri I Akkus, Gloria Caldito

**Affiliations:** 1 Department of Pulmonary, Critical Care and Sleep medicine, 1501 Kings Highway, Shreveport, LA 71103, USA; 2 Department of Surgery and Trauma, 1501 Kings Highway, Shreveport, LA 71103, USA; 3 Department of Cardiology, 1501 Kings Highway, Shreveport, LA 71103, USA; 4 Department of Biometry, 1501 Kings Highway, Shreveport, LA 71103, USA; 5 Louisiana State University Health Sciences Center-Shreveport, 1501 Kings Highway, Shreveport, LA 71103, USA

## Abstract

**Introduction:**

Ultrasonography is being increasingly utilized in acute care settings with expanding applications. Pneumothorax evaluation by ultrasonography is a fast, safe, easy and inexpensive alternative to chest radiographs. In this review, we provide a comprehensive analysis of the current literature comparing ultrasonography and chest radiography for the diagnosis of pneumothorax.

**Methods:**

We searched English-language articles in MEDLINE, EMBASE and Cochrane Library dealing with both ultrasonography and chest radiography for diagnosis of pneumothorax. In eligible studies that met strict inclusion criteria, we conducted a meta-analysis to evaluate the diagnostic accuracy of pleural ultrasonography in comparison with chest radiography for the diagnosis of pneumothorax.

**Results:**

We reviewed 601 articles and selected 25 original research articles for detailed review. Only 13 articles met all of our inclusion criteria and were included in the final analysis. One study used lung sliding sign alone, 12 studies used lung sliding and comet tail signs, and 6 studies searched for lung point in addition to the other two signs. Ultrasonography had a pooled sensitivity of 78.6% (95% CI, 68.1 to 98.1) and a specificity of 98.4% (95% CI, 97.3 to 99.5). Chest radiography had a pooled sensitivity of 39.8% (95% CI, 29.4 to 50.3) and a specificity of 99.3% (95% CI, 98.4 to 100). Our meta-regression and subgroup analyses indicate that consecutive sampling of patients compared to convenience sampling provided higher sensitivity results for both ultrasonography and chest radiography. Consecutive versus nonconsecutive sampling and trauma versus nontrauma settings were significant sources of heterogeneity. In addition, subgroup analysis showed significant variations related to operator and type of probe used.

**Conclusions:**

Our study indicates that ultrasonography is more accurate than chest radiography for detection of pneumothorax. The results support the previous investigations in this field, add new valuable information obtained from subgroup analysis, and provide accurate estimates for the performance parameters of both bedside ultrasonography and chest radiography for pneumothorax evaluation.

## Introduction

Chest ultrasonography (US) is gaining more attention in critical care and emergency medicine literature. US has been used recently for evaluation of pneumothorax and other lung pathologies. Several early trials [1-3] by Litchenstein *et al*. established the diagnostic signs of pneumothorax on US and showed a strong superiority in favor of US over chest radiography (CXR). Despite those and other cumulating original research evidence favoring ultrasonography, US remained underused. In fact, the most recent British thoracic society guidelines on pleural procedures and thoracic ultrasound stated that “The utility of thoracic ultrasound for diagnosing a pneumothorax is limited in hospital practice due to the ready availability of chest x-rays and conflicting data from published reports” [4]. During the years 2011 and 2012, an increasing number of original research publications compared US with CXR for pneumothorax evaluation, reflecting the expanding rule of US in evaluation of pneumothorax and the growing interest in it to replace CXR. Two meta-analyses [5,6] attempted for the first time to evaluate the diagnostic accuracy of US in comparison with CXR. In both studies, chest ultrasonography was a near-perfect test with very high sensitivity and specificity. Since the publication of those analyses, few other high-quality original research articles were published comparing the two tests for pneumothorax detection.

By using a regression-analysis model of the published literature, Lijmer *et al*. [7] showed that inclusion of a diseased population in meta-analyses of diagnostic tests overestimates the diagnostic odds ratio (DOR) by threefold and that using a different reference standard in those studies overestimates the DOR by at least twofold. DOR is defined as the odds for a positive test result in diseased persons relative to the odds of a positive result in nondiseased persons [7]. It is an indicator of test performance that is independent of the prevalence of the disease [7]. Studies with lower methodologic quality tend to include patients with known disease and apply the index test on them, which leads to overestimation of DOR by increasing the odds of having a positive test in diseased subjects. By using a different reference standard (in our analysis: obtaining CT scans preferentially on patients with pneumothorax compared with milder cases without pneumothorax) also overestimated the DOR by increasing the sensitivity and specificity of the index test and introduced possible selection biases to the study. Such studies were included in the previously published meta-analyses comparing the two tests.

We aimed to conduct an accurate meta-analysis of the available literature that included high-quality articles, and avoiding studies that evaluated populations with known pneumothorax and studies that used different verification methods. Additionally, we evaluated the studies for other possible sources of bias. We also intended to include the recent publications that were not included in previous analyses. Furthermore, we specifically planned to address the inherent heterogeneity found that could not be addressed in the previously published meta-analyses. We addressed issues related to operator, type of probe, and patient-population specifics. We believe that our study adds valuable information to the current literature in this field that could guide the application of pleural ultrasonography in the clinicians’ daily practices.

## Materials and methods

### Study design and data extraction

**Figure 1:**
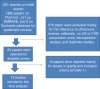
A diagram represents the review process and selection of included studies.

We performed a literature review and meta-analysis of published research articles evaluating the diagnostic accuracy of US in comparison with CXR. Original articles published in the English language up to March 2013 were searched in Medline, EMBASE, and the Cochrane Library. Our initial search was broad and included the following words: (“ultrasound” or “sonography” or “ultrasonography” or “radiography” or “chest film” or “chest radiograph”) and (“pneumothorax” or “aerothorax”) and (“sensitivity” and “specificity”). We noted a large number of articles published during the study, and so we performed three separate searches during the entire review process (December 2011, May 2012, and March 2013; EMBASE was accessed in January 2013). The number of articles and abstracts depicted in the diagram (Figure 1) represents the most-recent search. Bibliographies of the initially chosen articles and the previous meta-analyses were also subjected to a secondary hand search, and relevant articles were extracted. No institutional review board (IRB) approval or consents were needed for this review, as it evaluated published studies without individually identifiable human-subject information.

### Studies inclusion criteria

The inclusion criteria we used to select articles are as follows: (a) Original prospective blinded studies comparing the performance of US and CXR for pneumothorax diagnosis; (b) compared the two tests with one gold standard, the computed tomography (CT) scan of the chest; (c) avoided studies that included diseased populations (populations with known pneumothorax); (d) Described the diagnostic criteria for pneumothorax on US in clear details; And (e) met quality standards, as assessed by the 14-item Quality Assessment of Diagnostic Accuracy Studies (QUADAS2) [8] tool.

All patients included in our analysis had CT scans in addition to CXR and US examinations. If a study included patients with differential verifications, only patients with CT scans were included. If a study included patients studied by US for other conditions in addition to pneumothorax, only patients evaluated for pneumothorax were included.

### Review process

We identified 601 potential articles in our initial searches (see Figure 1). Authors (SA, AMY, and NIA) independently reviewed the abstracts of those articles and selected 25 articles in agreement for a detailed review. Disagreement was solved by discussion and with a second opinion from our institution’s statistician (GC). Out of the 25 articles, 12 were excluded (see Additional file 1: eTable S1 for list of excluded studies), mainly for not meeting more than 11 of the 14 QUADAS items (see Additional file 2), including (a) differential verification methods (two different reference standards); (b) blinding issues related to known pneumothorax; (c) too-long wait time (defined as waiting more than 6 hours) between index test and reference standard or vice versa; (d) insufficient details of the execution of the index test or the reference standard; (e) lack of either CT-scan verification CXR or US arm. One good-quality study [9] was excluded mainly because it included a significant number of patients (12 of 53) with severe clinical pneumothorax requiring chest-tube placement after US or CXR but without or before the gold standard, raising blinding issues and challenges in the statistical analysis. Another study [10] was excluded mainly because of insufficient data about CXR. Thirteen [11-23] studies passed all inclusion criteria and were included in the final analysis (Table 1).

**Table 1:** Characteristics of included studies

**Study**	**Publication year**	**Country**	**Operator**	**US signs**	**US probe**	**Sampling**	**Study subjects**
Donmez [11]	2012	Turkey	Radiologist	CT, LS, LP	Linear	NC	Trauma
Abbasi [12]	2012	Iran	Emergency physician	LS, CT	Linear	NC	Trauma
Hyacinthe^a^[13]	2012	France	Emergency physician	LS, CT, LP	Convex	NC	Trauma
Nandipati^b^[14]	2011	United States	Emergency physician	LS, CT	Linear	C	Trauma
Nagarsheth [15]	2011	United States	Surgeon	CT, LS	Convex and linear	NC	Trauma
Xirouchaki^a^[16]	2011	Greece	Intensivist	LS, CT, LP	Convex	NC	ICU
Brook [17]	2009	Israel	Radiologist	LS, CT	Convex	C	Trauma
Soldati [18]	2008	Italy	Emergency physician	LS, CT, LP	Convex	C	Trauma
Soldati [19]	2006	Italy	Emergency physician	LS, CT, LP	Convex	C	Trauma
Zhang [20]	2006	China	Emergency physician	LS, CT, LP	Convex, linear	NC	Trauma
Chung [21]	2005	South Korea	Radiologist	LS	Linear	C	Post-procedural
Kirkpatrick [22]^c^	2004	Canada and United States	Surgeon	LS, CT, PDS	Linear	NC	Trauma
Rowan [23]	2002	Canada	Radiologist	LS, CT	Linear	NC	Trauma

^a^Studies included multiple conditions; only pneumothorax patients were included.

^b^Only one intercostal space was examined in this study.

^c^Only patients with CT scans were included.

*C* consecutive sampling, *CT* comet tail (B-lines).

*LP* lung point, *LS* lung sliding, *NC* nonconsecutive (convenience) sampling. *PDS* power Doppler sign.

### Data synthesis

After extraction of data from the original studies, data were arranged in 2 × 2 tables expressing true positive (TP), false positive (FP), false negative (FN), and true negative (TN). In cases of uncertainty about data or the quality, the author was contacted (one case [14]). Five studies [11,16-18,22] reported their results considering hemithoraces as independent variables, whereas the others considered each patient as an independent variable. To overcome this challenge and maintain study weight estimates intact, we multiplied values in all cells of those studies by 2. In one study [21], four observations by independent operators were reported on each patient, considering the patient as a unit. In this case, we divided the results in all cells by 2. We evaluated all possible causes of heterogeneity and stratified studies according to the operator, ultrasound probe used, study subjects (trauma versus nontrauma), ultrasonographic signs assessed, as well as other possible sources of bias, including type of sampling (consecutive versus convenience sampling). We included these items as covariates in our data tables for meta-regression and subgroup analysis.

### Data analysis

We assumed that US and CXR have different accuracy when applied to different patient populations by different operators. For this reason, we used a random-effect model in our meta-analysis to calculate pooled sensitivity and specificity with corresponding 95% confidence intervals (CIs). Other data such as diagnostic odds ratio (DOR) and receiver operative curves (ROCs) were also obtained. We used Meta-DiSc [24], version 1.4 software (Ramon y Cajal Hospital, Madrid, Spain). We also used Review Manager 5.2, mainly to assess quality and risk of bias. Results of analysis using both software programs were identical. However, for this report, all data and graphs were obtained from the results of Meta-DiSc analysis, as it provided more information for reporting.

To explain the observed heterogeneity, we performed meta-regression and subgroup analyses, as applicable, using all observed covariates. Meta-regression is a regression analysis of the effects of covariates in relation to each other at the level of studies. The effect sizes were explained as diagnostic odds ratio (DOR) and relative diagnostic odds ratio (RDOR) in relation to the dependent variable of interest. To compare performance-parameter estimates (sensitivity, specificity, or DOR) for different diagnostic tests at 5% level, we used the calculated 95% confidence intervals (CIs) for a parameter estimate for the diagnostic tests being compared. In comparison with previous studies, we observed a significant difference in the estimated parameter for two values being compared if the 95% CI for the parameter of interest in our estimate did not include the parameter estimate in the other studies’ estimates or vice versa.

## Results

**Figure 2:**
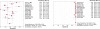
**Forest plot for sensitivity, specificity of CXR for the detection of pneumothorax.** Inconsistency (*I*^2^) describes the percentage heterogeneity across studies that are not due to chance. *I*^2^ can be calculated as *I*^2^ =100% 3 (*Q*^2^*df*)/*Q* (see Figure 4 legend for definition of Q). df = degree of freedom = number of studies-1.

**Figure 3:**
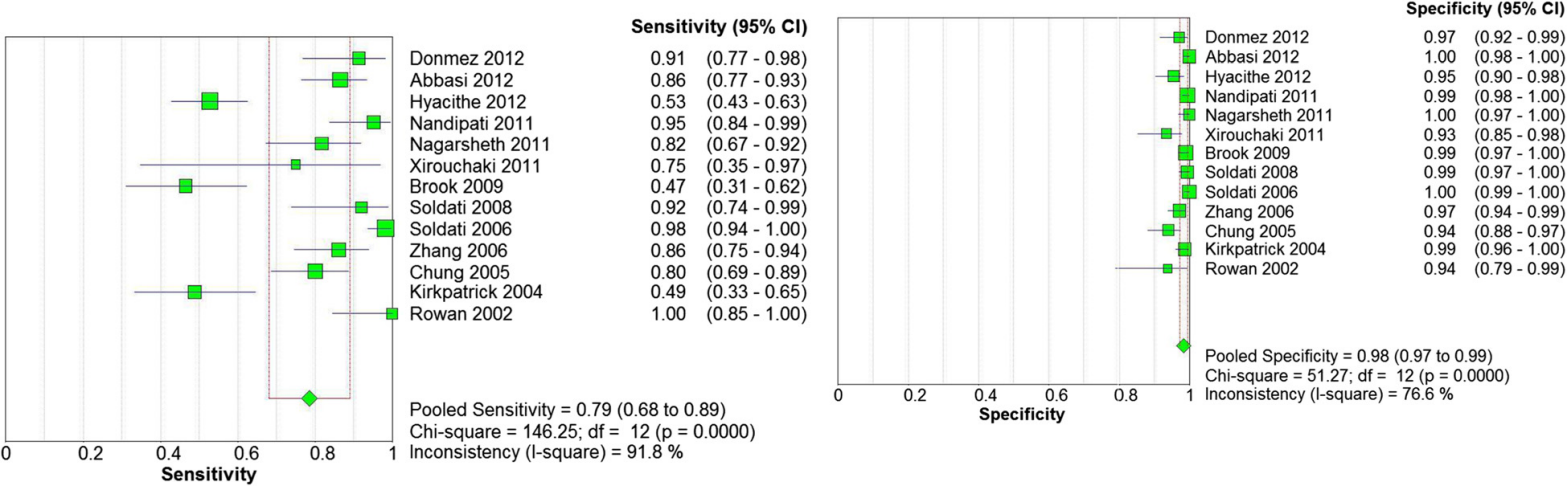
**Forest plot for sensitivity, specificity of US for detection of pneumothorax.** Inconsistency (*I*^2^) describes the percentage heterogeneity across studies that is not due to chance. (Refer to Figure 1 legend explanation of statistics).

**Figure 4:**
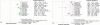
**Forest plot for diagnostic odds ratio (DOR) of US (left) and CXR (right).** DOR = positive likelihood ratio/negative likelihood ratio = TP × TN/FN × FP. Inconsistency (*I*^2^) describes the percentage heterogeneity across studies that is not due to chance. Tau-squared represents the amount of heterogeneity. Cochran *Q* is a statistic that represents a ratio of total observed variation to within-study error; it is usually computed by summing the squared deviations of each study’s estimate from the overall meta-analytic estimate.

**Figure 5:**
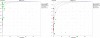
**Summary receiver operative curves for US (left) and CXR (right).** AUC, Area under the curve; SE, standard error.

From the 13 chosen studies (Table 1), we extracted the data from each study and conducted a random-effect model meta-analysis. In addition to quality assessment, we assessed for risk of bias and considered covariates that can affect heterogeneity. A total of 3,028 hemithoraces from 1,514 patients were included in the analysis. Our study revealed a clear superiority of US over CXR. The calculated pooled sensitivity for US and CXR were: 78.6% (95% CI, 68.1 to 98.1) and 39.8% (95% CI, 29.4 to 50.3), respectively. The pooled specificity for US and CXR were 98.4% (95% CI, 97.3 to 99.5) and 99.3% (95% CI, 98.4 to 100), respectively (Figures 2 and 3). The pooled DOR for US was 279.31 (95% CI, 106.29 to 733.94), whereas for CXR, the pooled DOR was 87.19 (95% CI, 33.44 to 229.34) (Figure 4). The summary receiver operating characteristic (sROC) curves for US and CXR are depicted in Figure 5. The areas under the curve (AUC) for US and CXR are 0.98 (SE, 0.0065) and 0.959 (SE, 0.014), respectively. Our results, as in previous meta-analyses [5,6], revealed a high degree of heterogeneity. Our analysis indicated that the observed variations were more likely between studies than within studies, as indicated by a high ratio of the variance of observed effects to total variance (denoted by the *I*^2^ statistic). We assessed possible sources of heterogeneity by performing meta-regression (and subgroup analyses when applicable) by using data on observed covariates for CXR (Additional file 1: eTable S2) and US (Additional file 1: eTable S3), and results are expressed as a relative diagnostic odds ratio (RDOR). By using subgroup analysis, type of sampling for patient selection and trauma/nontrauma setting were the significant covariates. The amount of variation in US was higher than that in CXR in the included studies (Tau-squared estimate, 2.2 versus 1.65; Figure 4).

For CXR, both consecutive sampling (RDOR = 6.77; 95% CI, 1.53 to 29.93; *P* = 0.017) and trauma settings (RDOR = 27.42; 95% CI, 5.36 to 140.31; *P* = 0.0013) were significantly associated with different results, with a higher DOR for consecutive sampling and trauma. Heterogeneity in CXR studies was fully explained by these factors with negligible residual T^2^ (Additional file 1: eTable S2). Subgroup analysis showed improvement in pooled sensitivity in consecutive-sampling studies to 49.7% (95% CI, 33.0 to 66.3). In contrast, in convenience sampling studies, the pooled sensitivity was significantly lower at 32.6% (95% CI, 20.7 to 44.5), whereas specificity remained comparable in all subgroups.

For US, both trauma settings (RDOR = 32.87; 95% CI, 2.42 to 447.03; *P* = 0.018) and consecutive sampling (RDOR = 21.99; 95% CI, 1.98 to 244.93; *P* = 0.021) were significant contributors to heterogeneity by using meta-regression analysis (Additional file 1: eTable S3). Subgroup analysis also showed that in consecutive sampling studies, the pooled sensitivity improved to 85.3% (95% CI, 68 to 100), whereas in nonconsecutive (convenience) sampling studies, the pooled sensitivity decreased to 73.6% (95% CI, 60.4 to 86.7). Studies that used the high-frequency linear array probe had a pooled sensitivity of 82.2% (95% CI, 68.8 to 95.5), whereas those using a convex array probe had a pooled sensitivity of 76% (95% CI, 59.8 to 92.3). Operator (radiologist versus others) was not a significant variable in our analysis. However, emergency physician-performed US had better sensitivity than nonemergency physicians-performed US (82.3% versus 72.8%). (Additional file 1 contains statistical tables for both CXR and US subanalyses).

In the discussion sections of the reviewed articles [11-23], few conditions were reported to cause false-positive results on US, including pleural adhesions, bullous emphysema, and main-stem intubation on the contralateral side. Subcutaneous emphysema and pleural calcifications were also reported to impede the US waves.

Ultrasonography time, when assessed [12,14,18,20,21], ranged between <2 minutes and 15 minutes. In all studies that evaluated test-performance time, except one [21], US time was significantly shorter than CXR time (mean, <5 minutes versus 10 to 15 minutes).

## Discussion

In the past 3 years, at least nine published original prospective research articles addressed the diagnostic accuracy of pleural ultrasonography for the diagnosis of pneumothorax, reflecting the growing interest in this valuable test as an alternative to CXR. Six of those articles [11-16] met our inclusion and quality criteria and, for the first time, were included in a meta-analysis. The pooled sensitivity of US in our study is the lowest of the previous two analyses [5,6] (78.6% versus 88% [5] and 90.9% [6]). The DOR reported by a previous meta-analysis [5] is 3.3 times higher than our result (993 with 95% CI, 333 to 2,957) indicating a threefold overestimation of the accuracy of the test and attesting to Lijmer’s previous estimates [7]. Both reported sensitivity and DOR are significantly different from those obtained in previous analyses, as shown from the reported confidence intervals.

We believe that our study provides better estimates of the test parameters because of the inclusion of a large number of good-quality standardized studies and patients (total of 1,514 patients) in the analysis. Our meta-analysis allowed, for the first time, the identification of significant sources of variation in the effect size among the included studies. It is the first to compare CT scan, US, and CXR in the same population on this large scale. On all counts US remains superior to CXR for detection of pneumothorax, even after controlling for possible sources of heterogeneity (the lowest US subgroup sensitivity was 73.6%). With positive test results, patients tested with US have greater odds of having an accurate diagnosis of pneumothorax than do those tested with CXR (DOR, 279.31 versus 87.19). The majority of studies included in our analysis were in trauma settings; this was expected, as an indication for CT scan of the chest is readily available in this setting. Our results indicated that a linear probe provided better sensitivity (82% versus 76%); this is likely because of the better views of the lung sliding sign obtained with this high-resolution probe.

In our study, emergency physicians performed better US than did nonemergency physicians (sensitivity, 82.3% versus 72.8%). This could be related to their early experience in thoracic US use as part of the eFAST (Extended Focused Assessment with Sonography for Trauma) that emphasized the importance of training and experience in this operator-dependent test.

Our study is not without limitations. Despite meticulous efforts to explain possible causes of heterogeneity, we were unable to account for some minor sources, especially on the US analysis, which had some minimal residual heterogeneity after meta-regression (*T*^2^ = 0.2; Additional file 1: eTable S3). We did not evaluate our meta-analysis for publication bias. We kept our search very broad initially to overcome this issue but included all studies that performed US and CXR as well as CT scan as the gold standard to maintain accuracy and avoid overestimates of diagnostic accuracy.

Studies published in languages other than English, with the exception of one [10], were not identified in our search and were not included. This probably has a negligible effect. CXR was performed in supine position in the vast majority of our study patients, and this may have underestimated test performance. In nontrauma studies [16,21], in which a large number of patients had semierect CXR, the sensitivity only increased to 42.3% (95% CI, 14.3 to 70.3). A previous meta-analysis [5] included studies of CXR alone to neutralize the effect of patient position, and their reported sensitivity was only 52%.

As stated earlier, most of our included studies were in trauma/emergency department settings. This was mainly the result of including studies that compared both tests with the gold standard (CT scan of the chest). It should be noted that the severity of trauma was not assessed in the majority of those studies. The consecutive-sampling studies (which showed higher sensitivity) may have allowed a wide spectrum of patients to be included in those studies. Furthermore, most pneumothoraces missed by CXR were occult and detected only by US and CT scans. In one study [22] that reported injury severity score (ISS), occult pneumothorax was 8 times more likely to be present in patients with ISS >16. Studies comparing US and CXR with CT scans in evaluation of postbronchoscopy pneumothorax, for example, would expose patients to unnecessary radiation from CT scan and would be unethical. A small pneumothorax might not require invasive treatment, but it is important to recognize, in certain clinical situations, such as positive-pressure ventilation and air transportation.

It is important to note that the test characteristics are only part of the assessment of a diagnostic test performance, and the value of any test ultimately lies in its effects on patient outcome. Other important factors such as potential of harm as a consequence of the test (in our case, possible exposure to unnecessary procedures to treat a small pneumothorax or exposure to ionizing radiation), physician’s perception and confidence in test results, as well as the ability to make treatment decisions based on test results were not addressed in our study [25]. Future research can be designed to address the downstream effects of two separate testing strategies specifically for pneumothorax: one that uses CXR and another for US. Possible outcome measures are number of invasive procedures and subsequent tests resulting from the index test, and total condition-related cost of care. We expect US to be safer, more convenient, more cost effective, and to outperform CXR in most aspects.

## Conclusion

Despite the lower sensitivity and lower DOR found in our analysis, US remains much more sensitive than CXR for identification of pneumothorax. Our analysis supports the available evidence in favor of ultrasonography over chest radiography and provides an objective assessment of the diagnostic performance of both tests in the well-designed published studies that we included in our meta-analysis. Our analysis identifies several important factors that increase the accuracy of US in detection of pneumothorax, including operator experience, patient population, and the type of probe used.

## Key messages

• Pleural ultrasound is less sensitive than previously reported but remains superior to chest radiograph for detection of pneumothorax in the trauma and critical care populations.

• Training and familiarity with bedside ultrasound techniques may provide better accuracy, as appeared with the emergency physician performance.

• High-resolution linear probe gives higher accuracy for the sliding pleura sign.

• Ultrasound is convenient, is a readily available bedside procedure, is easy to learn, is accurate for diagnosing pneumothorax, and avoids patient exposure to ionizing radiation.

## Abbreviations

CXR: Chest radiography; DOR: Diagnostic odds ratio; eFAST: Extended focused assessment with sonography for trauma; RDOR: Relative diagnostic odds ratio; sROC: Summary receiver operating characteristic; US: Ultrasonography.

## Competing interests

The authors declare that they have no competing interests with this scientific work.

## Authors’ contribution

SA is responsible for the integrity of this work from inception to manuscript preparation, And contributed to study design, studies selection, quality assessment and records review, data synthesis, data analysis, and manuscript composition. AY contributed to the review process by providing review of 50% of the search records and manuscript review. NIA contributed to the review process by providing review of 50% of the search records and manuscript review. GC contributed to study by providing statistical advice during the review process, final studies selection, and manuscript review. All authors read and approved the final manuscript.

## Authors’ information

Saadah Alrajab, MD, MPH; department of pulmonary, critical care and sleep medicine. Louisiana State University Health Sciences center and Cogent-The Intensivist Group, St.Bernardine Medical Center (California). Asser M Ypussef, MD; department of surgery and trauma. Louisiana State University Health Sciences center. Nuri I Akkus, MD; department of cardiology. Louisiana State University Health Sciences center. Gloria Caldito, PhD; department of biometry. Louisiana State University Health Sciences center.

## Supplementary Material

Additional file 1 Contains details of the excluded studies, statistical tables for main analysis, and subgroup analyses. Click here for file

Additional file 2 Contains QUADAS quality-assessment items for the included studies. Click here for file
