# Supplementary material for: Pleural ultrasonography versus chest radiography for the diagnosis of pneumothorax: review of the literature and meta-analysis
Source: Crit Care. 2013 Sep 23;17(5):R208. doi: 10.1186/cc13016 (PMC4057340; doi:10.1186/cc13016)
Supplement: Additional file 1 — Contains details of the excluded studies, statistical tables for main analysis, and subgroup analyses. [file cc13016-S1.pdf]

<u>Additional file-1</u>: Contains details of the excluded studies, statistical tables for main analysis and subgroup analyses.

eTable-1: list of excluded studies

Study	Comment
1: Jalli R, Sefidbakht S, Jafari SH. Value of ultrasound in diagnosis of pneumothorax: a prospective study. Emerg Radiol. 2013 Apr;20(2):131-4.	- Unacceptably long time between index test and reference standard (up to 48h).<br>- High risk of bias<br>- Insufficient details of index test
2: Mennicke M, Gulati K, Oliva I, Goldflam K, Skali H, Ledbetter S, Platz E. Anatomical distribution of traumatic pneumothoraces on chest computed tomography: implications for ultrasound screening in the ED. Am J Emerg Med. 2012 Sep;30(7):1025-31	- No US arm
3: Zanobetti M, Poggioni C, Pini R. Can chest ultrasonography replace standard chest radiography for evaluation of acute dyspnea in the ED? Chest. 2011 May;139(5):1140-7.	- High risk of bias<br>- Differential verification<br>- Reference standard was not independent of the index test.
4: Vezzani A, Brusasco C, Palermo S, Launo C, Mergoni M, Corradi F. Ultrasound localization of central vein catheter and detection of postprocedural pneumothorax: an alternative to chest radiography. Crit Care Med. 2010 Feb;38(2):533-8.	- No CT scan, CXR was considered a reference standard<br>- Few pneumothorax patients
5: Galbois A, Ait-Oufella H, Baudel JL, Kofman T, Bottero J, Viennot S, Rabate C, Jabbouri S, Bouzeman A, Guidet B, Offenstadt G, Maury E. Pleural ultrasound compared with chest radiographic detection of pneumothorax resolution after drainage. Chest. 2010 Sep; 138(3):648-55.	- Evaluated patients with known pneumothorax<br>- Differential verification<br>- Possible blinding and methodological issues
6: Sartori S, Tombesi P, Trevisani L, Nielsen I, Tassinari D, Abbasciano V. Accuracy of transthoracic sonography in detection of pneumothorax after sonographically guided lung biopsy: prospective comparison with chest radiography. AJR Am J Roentgenol. 2007 Jan;188(1):37-41.	- Differential verification<br>- Blinding issues
7: Garofalo G, Busso M, Perotto F, De Pascale A, Fava C. Ultrasound diagnosis of pneumothorax. Radiol Med. 2006 Jun;111(4):516-25. Epub 2006 May 29.	- Insufficient CXR data<br>- Unclear if all patients received CXR or not.<br>- Possible selection bias
8: Blaivas M , Lyon M , Duggal S . A prospective comparison of supine chest radiography and bedside ultrasound for thediagnosis of traumatic pneumothorax . *Acad Emerg Med* . 2005 ; 12 (9): 844 - 849 .	- Possible blinding issues<br>- See text
9: Reissig A, Kroegel C. [Sonographic diagnosis of post-interventional pneumothorax and hydropneumothorax--prospective study of 100 patients]. Praxis (Bern 1994). 2006 Apr 19;95(16):617-24.	- Differential verification<br>- Partial verification<br>- Reference standard was not independent of the index test.
10: Lichtenstein D, Mezière G, Biderman P, Gepner A. The "lung point": an ultrasound sign specific to pneumothorax. Intensive Care Med. 2000 Oct;26(10):1434-40.	- Partial verification<br>- Possible blinding issues<br>- Patients with known pneumothorax
11: Lichtenstein D, Mezière G, Biderman P, Gepner A. The comet-tail artifact: an ultrasound sign ruling out pneumothorax. Intensive Care Med. 1999 Apr;25(4):383-8.	- Partial verification<br>- Possible blinding issues<br>- Patients with known pneumothorax
12: Lichtenstein DA, Menu Y. A bedside ultrasound sign ruling out pneumothorax in the critically ill. Lung sliding. Chest. 1995 Nov;108(5):1345-8.	- Partial verification<br>- Possible blinding issues<br>- Patients with known pneumothorax

eTable-2

CXR - Meta-Regression (Inverse Variance weights)

Variable	Coeff.	Std. Err.	p - value	RDOR	[95%CI]
**Traum/Non-trauma**	3.311	0.7217	0.0013	27.42	(5.36-140.31)
**Consecutive/non-consecutive**	1.912	0.6572	0.0173	6.77	(1.53-29.93)

Tau-squared estimate = 0.0000 (Convergence is achieved after 1 iterations)
Maximum Likelihood estimation (ML).
No. studies = 13
RDOR= Relative Diagnostic Odds Ratio

eTable-3

US-Meta-Regression (Inverse Variance weights)

Variable	Coeff.	Std. Err.	p - value	RDOR	[95%CI]
**Operator ER physician vs others**	-1.296	1.1082	0.2950	0.27	(0.02-4.73)
**Operator Radiologist vs others**	-2.719	1.0912	0.0551	0.07	(0.00-1.09)
**Trauma patients vs non-trauma**	3.493	1.0154	0.0184	32.87	(2.42-447.03)
**Consecutive vs Non-consecutive**	3.091	0.9376	0.0216	21.99	(1.98-244.93)
**Linear array probe vs convex**	0.799	0.8418	0.3861	2.22	(0.26-19.35)
**Lung point evaluated or not**	0.119	1.0555	0.9145	1.13	(0.07-16.99)

Tau-squared estimate = 0.2671 (Convergence is achieved after 15 iterations)
Maximum Likelihood estimation (ML).
No. studies = 13
RDOR= Relative Diagnostic Odds Ratio

<u>Sensitivity and specificity of all CXR studies</u>

Summary Sensitivity

Study	Sen	[95% Conf. Iterval.]		TP/(TP+FN)	TN/(TN+FP)
Donmez 2012	0.686	0.507	- 0.831	24/35	96/101
Abbasi 2012	0.486	0.369	- 0.606	36/74	218/218
Hyacithe 2012	0.189	0.119	- 0.276	20/106	132/132
Nandipati 2011	0.789	0.627	- 0.904	30/38	368/370
Nagarsheth 2011	0.318	0.186	- 0.476	14/44	114/114
Xirouchaki 2011	0.000	0.000	- 0.369	0/8	75/76
Brook 2009	0.163	0.068	- 0.307	7/43	295/295
Soldati 2008	0.520	0.313	- 0.722	13/25	193/193
Soldati 2006	0.536	0.439	- 0.630	60/112	260/260
Zhang 2006	0.276	0.167	- 0.409	16/58	212/212
Chung 2005	0.471	0.351	- 0.594	33/70	116.5/124
Kirkpatrick 2004	0.209	0.100	- 0.360	9/43	222/223
Rowan 2002	0.364	0.172	- 0.593	8/22	32/32
**Pooled Sen**	**0.398**	**0.294**	**- 0.503**		

Heterogeneity chi-squared = 103.07 (d.f.= 12) p = 0.000
Inconsistency (I-square) = 88.4 %
No. studies = 13.
Pooled Confidence Interval corrected for overdispersion

Summary Specificity

Study	Spe	[95% Conf. Iterval.]		TP/(TP+FN)	TN/(TN+FP)
Donmez 2012	0.950	0.888	- 0.984	24/35	96/101
Abbasi 2012	1.000	0.983	- 1.000	36/74	218/218
Hyacithe 2012	1.000	0.972	- 1.000	20/106	132/132
Nandipati 2011	0.995	0.981	- 0.999	30/38	368/370
Nagarsheth 2011	1.000	0.968	- 1.000	14/44	114/114
Xirouchaki 2011	0.987	0.929	- 1.000	0/8	75/76
Brook 2009	1.000	0.988	- 1.000	7/43	295/295
Soldati 2008	1.000	0.981	- 1.000	13/25	193/193
Soldati 2006	1.000	0.986	- 1.000	60/112	260/260
Zhang 2006	1.000	0.983	- 1.000	16/58	212/212
Chung 2005	0.940	0.882	- 0.974	33/70	116.5/124
Kirkpatrick 2004	0.996	0.975	- 1.000	9/43	222/223
Rowan 2002	1.000	0.891	- 1.000	8/22	32/32
**Pooled Spe**	**0.993**	**0.984**	**- 1.000**		

Heterogeneity chi-squared = 51.77 (d.f.= 12) p = 0.000
Inconsistency (I-square) = 76.8 %
No. studies = 13.
Pooled Confidence Interval corrected for overdispersion

Sensitivity and specificity of all US studies

Summary Sensitivity

Study	Sen	[95% Conf. Iterval.]		TP/(TP+FN)	TN/(TN+FP)
Donmez 2012	0.914	0.769	- 0.982	32/35	98/101
Abbasi 2012	0.865	0.765	- 0.933	64/74	218/218
Hyacithe 2012	0.528	0.429	- 0.626	56/106	126/132
Nandipati 2011	0.952	0.838	- 0.994	40/42	364/366
Nagarsheth 2011	0.818	0.673	- 0.918	36/44	114/114
Xirouchaki 2011	0.750	0.349	- 0.968	6/8	71/76
Brook 2009	0.465	0.312	- 0.623	20/43	292/295
Soldati 2008	0.920	0.740	- 0.990	23/25	192/193
Soldati 2006	0.982	0.937	- 0.998	110/112	260/260
Zhang 2006	0.862	0.746	- 0.939	50/58	206/212
Chung 2005	0.800	0.687	- 0.886	56/70	116.5/124
Kirkpatrick 2004	0.488	0.333	- 0.645	21/43	220/223
Rowan 2002	1.000	0.846	- 1.000	22/22	30/32
**Pooled Sen**	**0.786**	**0.681**	**- 0.891**		

Heterogeneity chi-squared = 146.25 (d.f.= 12) p = 0.000
Inconsistency (I-square) = 91.8 %
No. studies = 13.
Pooled Confidence Interval corrected for overdispersion

Summary Specificity

Study	Spe	[95% Conf. Iterval.]		TP/(TP+FN)	TN/(TN+FP)
Donmez 2012	0.970	0.916	- 0.994	32/35	98/101
Abbasi 2012	1.000	0.983	- 1.000	64/74	218/218
Hyacithe 2012	0.955	0.904	- 0.983	56/106	126/132
Nandipati 2011	0.995	0.980	- 0.999	40/42	364/366
Nagarsheth 2011	1.000	0.968	- 1.000	36/44	114/114
Xirouchaki 2011	0.934	0.853	- 0.978	6/8	71/76
Brook 2009	0.990	0.971	- 0.998	20/43	292/295
Soldati 2008	0.995	0.971	- 1.000	23/25	192/193
Soldati 2006	1.000	0.986	- 1.000	110/112	260/260
Zhang 2006	0.972	0.939	- 0.990	50/58	206/212
Chung 2005	0.940	0.882	- 0.974	56/70	116.5/124
Kirkpatrick 2004	0.987	0.961	- 0.997	21/43	220/223
Rowan 2002	0.938	0.792	- 0.992	22/22	30/32
**Pooled Spe**	**0.984**	**0.973**	**- 0.995**		

Heterogeneity chi-squared = 51.27 (d.f.= 12) p = 0.000
Inconsistency (I-square) = 76.6 %
No. studies = 13.
Pooled Confidence Interval corrected for overdispersion

Sensitivity and specificity of consecutive sampling CXR studies

Summary Sensitivity

Study	Sen	[95% Conf. Iterval.]		TP/(TP+FN)	TN/(TN+FP)
Nandipati 2011	0.789	0.627	- 0.904	30/38	368/370
Brook 2009	0.163	0.068	- 0.307	7/43	295/295
Soldati 2008	0.520	0.313	- 0.722	13/25	193/193
Soldati 2006	0.536	0.439	- 0.630	60/112	260/260
Chung 2005	0.471	0.351	- 0.594	33/70	116.5/124
**Pooled Sen**	**0.497**	**0.330**	**- 0.663**		

Heterogeneity chi-squared = 35.80 (d.f.= 4) p = 0.000
Inconsistency (I-square) = 88.8 %
No. studies = 5.
Pooled Confidence Interval corrected for overdispersion

Summary Specificity

Study	Spe	[95% Conf. Iterval.]		TP/(TP+FN)	TN/(TN+FP)
Nandipati 2011	0.995	0.981	- 0.999	30/38	368/370
Brook 2009	1.000	0.988	- 1.000	7/43	295/295
Soldati 2008	1.000	0.981	- 1.000	13/25	193/193
Soldati 2006	1.000	0.986	- 1.000	60/112	260/260
Chung 2005	0.940	0.882	- 0.974	33/70	116.5/124
**Pooled Spe**	**0.992**	**0.975**	**- 1.000**		

Heterogeneity chi-squared = 30.03 (d.f.= 4) p = 0.000
Inconsistency (I-square) = 86.7 %
No. studies = 5.
Pooled Confidence Interval corrected for overdispersion

Sensitivity and specificity of CXR convenience sampling studies

Summary Sensitivity

Study	Sen	[95% Conf. Iterval.]		TP/(TP+FN)	TN/(TN+FP)
Donmez 2012	0.686	0.507	- 0.831	24/35	96/101
Abbasi 2012	0.486	0.369	- 0.606	36/74	218/218
Hyacithe 2012	0.189	0.119	- 0.276	20/106	132/132
Nagarsheth 2011	0.318	0.186	- 0.476	14/44	114/114
Xirouchaki 2011	0.000	0.000	- 0.369	0/8	75/76
Zhang 2006	0.276	0.167	- 0.409	16/58	212/212
Kirkpatrick 2004	0.209	0.100	- 0.360	9/43	222/223
Rowan 2002	0.364	0.172	- 0.593	8/22	32/32
**Pooled Sen**	**0.326**	**0.207**	**- 0.445**		

Heterogeneity chi-squared = 47.11 (d.f.= 7) p = 0.000
Inconsistency (I-square) = 85.1 %
No. studies =8.
Pooled Confidence Interval corrected for overdispersion

Summary Specificity

Study	Spe	[95% Conf. Iterval.]		TP/(TP+FN)	TN/(TN+FP)
Donmez 2012	0.950	0.888	- 0.984	24/35	96/101
Abbasi 2012	1.000	0.983	- 1.000	36/74	218/218
Hyacithe 2012	1.000	0.972	- 1.000	20/106	132/132
Nagarsheth 2011	1.000	0.968	- 1.000	14/44	114/114
Xirouchaki 2011	0.987	0.929	- 1.000	0/8	75/76
Zhang 2006	1.000	0.983	- 1.000	16/58	212/212
Kirkpatrick 2004	0.996	0.975	- 1.000	9/43	222/223
Rowan 2002	1.000	0.891	- 1.000	8/22	32/32
**Pooled Spe**	**0.994**	**0.983**	**- 1.000**		

Heterogeneity chi-squared = 21.59 (d.f.= 7) p = 0.003
Inconsistency (I-square) = 67.6 %
No. studies = 8.
Pooled Confidence Interval corrected for overdispersion

Sensitivity and specificity of non-trauma CXR studies

Summary Sensitivity

Study	Sen	[95% Conf. Iterval.]	TP/(TP+FN)	TN/(TN+FP)
Xirouchaki 2011	0.000	0.000 - 0.369	0/8	75/76
Chung 2005	0.471	0.351 - 0.594	33/70	116.5/124
**Pooled Sen**	**0.423**	**0.143 - 0.703**		

Heterogeneity chi-squared = 9.47 (d.f.= 1) p = 0.002
Inconsistency (I-square) = 89.4 %
No. studies = 2.
Pooled Confidence Interval corrected for overdispersion

Summary Specificity

Study	Spe	[95% Conf. Iterval.]	TP/(TP+FN)	TN/(TN+FP)
Xirouchaki 2011	0.987	0.929 - 1.000	0/8	75/76
Chung 2005	0.940	0.882 - 0.974	33/70	116.5/124
**Pooled Spe**	**0.958**	**0.912 - 1.000**		

Heterogeneity chi-squared = 3.06 (d.f.= 1) p = 0.080
Inconsistency (I-square) = 67.3 %
No. studies = 2.
Pooled Confidence Interval corrected for overdispersion

Sensitivity and specificity of US consecutive sampling studies

Summary Sensitivity

Study	Sen	[95% Conf. Iterval.]		TP/(TP+FN)	TN/(TN+FP)
Nandipati 2011	0.952	0.838	- 0.994	40/42	364/366
Brook 2009	0.465	0.312	- 0.623	20/43	292/295
Soldati 2008	0.920	0.740	- 0.990	23/25	192/193
Soldati 2006	0.982	0.937	- 0.998	110/112	260/260
Chung 2005	0.800	0.687	- 0.886	56/70	116.5/124
**Pooled Sen**	**0.853**	**0.680**	**- 1.000**		

Heterogeneity chi-squared = 64.53 (d.f.= 4) p = 0.000
Inconsistency (I-square) = 93.8 %
No. studies = 5.
Pooled Confidence Interval corrected for overdispersion

Summary Specificity

Study	Spe	[95% Conf. Iterval.]		TP/(TP+FN)	TN/(TN+FP)
Nandipati 2011	0.995	0.980	- 0.999	40/42	364/366
Brook 2009	0.990	0.971	- 0.998	20/43	292/295
Soldati 2008	0.995	0.971	- 1.000	23/25	192/193
Soldati 2006	1.000	0.986	- 1.000	110/112	260/260
Chung 2005	0.940	0.882	- 0.974	56/70	116.5/124
**Pooled Spe**	**0.989**	**0.973**	**- 1.000**		

Heterogeneity chi-squared = 21.39 (d.f.= 4) p = 0.000
Inconsistency (I-square) = 81.3 %
No. studies = 5.
Pooled Confidence Interval corrected for overdispersion

Sensitivity and specificity of US convenience sampling studies

Summary Sensitivity

Study	Sen	[95% Conf. Iterval.]		TP/(TP+FN)	TN/(TN+FP)
Donmez 2012	0.914	0.769	- 0.982	32/35	98/101
Abbasi 2012	0.865	0.765	- 0.933	64/74	218/218
Hyacithe 2012	0.528	0.429	- 0.626	56/106	126/132
Nagarsheth 2011	0.818	0.673	- 0.918	36/44	114/114
Xirouchaki 2011	0.750	0.349	- 0.968	6/8	71/76
Zhang 2006	0.862	0.746	- 0.939	50/58	206/212
Kirkpatrick 2004	0.488	0.333	- 0.645	21/43	220/223
Rowan 2002	1.000	0.846	- 1.000	22/22	30/32
**Pooled Sen**	**0.736**	**0.604**	**- 0.867**		

Heterogeneity chi-squared = 67.76 (d.f.= 7) p = 0.000
Inconsistency (I-square) = 89.7 %
No. studies = 8.
Pooled Confidence Interval corrected for overdispersion

Summary Specificity

Study	Spe	[95% Conf. Iterval.]		TP/(TP+FN)	TN/(TN+FP)
Donmez 2012	0.970	0.916	- 0.994	32/35	98/101
Abbasi 2012	1.000	0.983	- 1.000	64/74	218/218
Hyacithe 2012	0.955	0.904	- 0.983	56/106	126/132
Nagarsheth 2011	1.000	0.968	- 1.000	36/44	114/114
Xirouchaki 2011	0.934	0.853	- 0.978	6/8	71/76
Zhang 2006	0.972	0.939	- 0.990	50/58	206/212
Kirkpatrick 2004	0.987	0.961	- 0.997	21/43	220/223
Rowan 2002	0.938	0.792	- 0.992	22/22	30/32
**Pooled Spe**	**0.977**	**0.962**	**- 0.993**		

Heterogeneity chi-squared = 24.92 (d.f.= 7) p = 0.001
Inconsistency (I-square) = 71.9 %
No. studies = 8.
Pooled Confidence Interval corrected for overdispersion

US studies: Linear array probe sensitivity and specificity

Summary Sensitivity

Study	Sen	[95% Conf. Iterval.]		TP/(TP+FN)	TN/(TN+FP)
Donmez 2012	0.914	0.769	- 0.982	32/35	98/101
Abbasi 2012	0.865	0.765	- 0.933	64/74	218/218
Nandipati 2011	0.952	0.838	- 0.994	40/42	364/366
Chung 2005	0.800	0.687	- 0.886	56/70	116.5/124
Kirkpatrick 2004	0.488	0.333	- 0.645	21/43	220/223
Rowan 2002	1.000	0.846	- 1.000	22/22	30/32
**Pooled Sen**	**0.822**	**0.688**	**- 0.955**		

Heterogeneity chi-squared = 43.36 (d.f.= 5) p = 0.000
Inconsistency (I-square) = 88.5 %
No. studies = 6.
Pooled Confidence Interval corrected for overdispersion

Summary Specificity

Study	Spe	[95% Conf. Iterval.]		TP/(TP+FN)	TN/(TN+FP)
Donmez 2012	0.970	0.916	- 0.994	32/35	98/101
Abbasi 2012	1.000	0.983	- 1.000	64/74	218/218
Nandipati 2011	0.995	0.980	- 0.999	40/42	364/366
Chung 2005	0.940	0.882	- 0.974	56/70	116.5/124
Kirkpatrick 2004	0.987	0.961	- 0.997	21/43	220/223
Rowan 2002	0.938	0.792	- 0.992	22/22	30/32
**Pooled Spe**	**0.984**	**0.966**	**- 1.000**		

Heterogeneity chi-squared = 23.25 (d.f.= 5) p = 0.000
Inconsistency (I-square) = 78.5 %
No. studies = 6.
Pooled Confidence Interval corrected for overdispersion

US studies: Convex array probe sensitivity and specificity

Summary Sensitivity

Study	Sen	[95% Conf. Iterval.]		TP/(TP+FN)	TN/(TN+FP)
Hyacithe 2012	0.528	0.429	- 0.626	56/106	126/132
Nagarsheth 2011	0.818	0.673	- 0.918	36/44	114/114
Xirouchaki 2011	0.750	0.349	- 0.968	6/8	71/76
Brook 2009	0.465	0.312	- 0.623	20/43	292/295
Soldati 2008	0.920	0.740	- 0.990	23/25	192/193
Soldati 2006	0.982	0.937	- 0.998	110/112	260/260
Zhang 2006	0.862	0.746	- 0.939	50/58	206/212
**Pooled Sen**	**0.760**	**0.598**	**- 0.923**		

Heterogeneity chi-squared = 99.09 (d.f.= 6) p = 0.000
Inconsistency (I-square) = 93.9 %
No. studies = 7.
Pooled Confidence Interval corrected for overdispersion

Summary Specificity

Study	Spe	[95% Conf. Iterval.]		TP/(TP+FN)	TN/(TN+FP)
Hyacithe 2012	0.955	0.904	- 0.983	56/106	126/132
Nagarsheth 2011	1.000	0.968	- 1.000	36/44	114/114
Xirouchaki 2011	0.934	0.853	- 0.978	6/8	71/76
Brook 2009	0.990	0.971	- 0.998	20/43	292/295
Soldati 2008	0.995	0.971	- 1.000	23/25	192/193
Soldati 2006	1.000	0.986	- 1.000	110/112	260/260
Zhang 2006	0.972	0.939	- 0.990	50/58	206/212
**Pooled Spe**	**0.984**	**0.968**	**- 0.999**		

Heterogeneity chi-squared = 28.03 (d.f.= 6) p = 0.000
Inconsistency (I-square) = 78.6 %
No. studies = 7.
Pooled Confidence Interval corrected for overdispersion

Emergency physician-performed US: sensitivity and specificity

Summary Sensitivity

Study	Sen	[95% Conf. Iterval.]		TP/(TP+FN)	TN/(TN+FP)
Abbasi 2012	0.865	0.765	- 0.933	64/74	218/218
Hyacithe 2012	0.528	0.429	- 0.626	56/106	126/132
Nandipati 2011	0.952	0.838	- 0.994	40/42	364/366
Soldati 2008	0.920	0.740	- 0.990	23/25	192/193
Soldati 2006	0.982	0.937	- 0.998	110/112	260/260
Zhang 2006	0.862	0.746	- 0.939	50/58	206/212
**Pooled Sen**	**0.823**	**0.667**	**- 0.978**		

Heterogeneity chi-squared = 88.07 (d.f.= 5) p = 0.000
Inconsistency (I-square) = 94.3 %
No. studies = 6.
Pooled Confidence Interval corrected for overdispersion

Summary Specificity

Study	Spe	[95% Conf. Iterval.]		TP/(TP+FN)	TN/(TN+FP)
Abbasi 2012	1.000	0.983	- 1.000	64/74	218/218
Hyacithe 2012	0.955	0.904	- 0.983	56/106	126/132
Nandipati 2011	0.995	0.980	- 0.999	40/42	364/366
Soldati 2008	0.995	0.971	- 1.000	23/25	192/193
Soldati 2006	1.000	0.986	- 1.000	110/112	260/260
Zhang 2006	0.972	0.939	- 0.990	50/58	206/212
**Pooled Spe**	**0.989**	**0.976**	**- 1.000**		

Heterogeneity chi-squared = 24.74 (d.f.= 5) p = 0.000
Inconsistency (I-square) = 79.8 %
No. studies = 6.
Pooled Confidence Interval corrected for overdispersion

Non-Emergency physician-performed US: sensitivity and specificity

Summary Sensitivity

Study	Sen	[95% Conf. Iterval.]		TP/(TP+FN)	TN/(TN+FP)
Donmez 2012	0.914	0.769	- 0.982	32/35	98/101
Nagarsheth 2011	0.818	0.673	- 0.918	36/44	114/114
Xirouchaki 2011	0.750	0.349	- 0.968	6/8	71/76
Brook 2009	0.465	0.312	- 0.623	20/43	292/295
Chung 2005	0.800	0.687	- 0.886	56/70	116.5/124
Kirkpatrick 2004	0.488	0.333	- 0.645	21/43	220/223
Rowan 2002	1.000	0.846	- 1.000	22/22	30/32
**Pooled Sen**	**0.728**	**0.581**	**- 0.876**		

Heterogeneity chi-squared = 49.78 (d.f.= 6) p = 0.000
Inconsistency (I-square) = 87.9 %
No. studies = 7.
Pooled Confidence Interval corrected for overdispersion

Summary Specificity

Study	Spe	[95% Conf. Iterval.]		TP/(TP+FN)	TN/(TN+FP)
Donmez 2012	0.970	0.916	- 0.994	32/35	98/101
Nagarsheth 2011	1.000	0.968	- 1.000	36/44	114/114
Xirouchaki 2011	0.934	0.853	- 0.978	6/8	71/76
Brook 2009	0.990	0.971	- 0.998	20/43	292/295
Chung 2005	0.940	0.882	- 0.974	56/70	116.5/124
Kirkpatrick 2004	0.987	0.961	- 0.997	21/43	220/223
Rowan 2002	0.938	0.792	- 0.992	22/22	30/32
**Pooled Spe**	**0.976**	**0.958**	**- 0.994**		

Heterogeneity chi-squared = 20.26 (d.f.= 6) p = 0.002
Inconsistency (I-square) = 70.4 %
No. studies = 7.
Pooled Confidence Interval corrected for overdispersion
