# Supplementary material for: Pleural ultrasonography versus chest radiography for the diagnosis of pneumothorax: review of the literature and meta-analysis
Source: Crit Care. 2013 Sep 23;17(5):R208. doi: 10.1186/cc13016 (PMC4057340; doi:10.1186/cc13016)
Supplement: Additional file 2 — Contains QUADAS quality-assessment items for the included studies. [file cc13016-S2.pdf]

	QUADAS Items	Donmez[11]	Abbasi[12]	Hyacinthe[13]	Nandipati[14]	Nagarsheth[15]	Xirouchaki[16]
1)	Was the spectrum of patients representative of the patients who will receive the test in practice? (spectrum composition)	Y	Y	Y	Y	Y	Y
2)	Were selection criteria clearly described? (selection criteria)	Y	Y	Y	Y	Y	Y
3)	*Is the reference standard likely to correctly classify the target condition? (reference standard)*	Y	Y	Y	Y	Y	Y
4)	Is the time period between reference standard and index test short enough to be reasonably sure that the target condition did not change between the two tests? (disease progression bias)	Y	U	Y	Y	U	Y
5)	Did the whole sample or a random selection of the sample, receive verification using a reference standard of diagnosis? (partial verification)	Y	Y	Y	Y	Y	Y
6)	Did patients receive the same reference standard regardless of the index test result? (differential verification)	Y	Y	Y	Y	Y	Y
7)	Was the reference standard independent of the index test (i.e. the index test did not form part of the reference standard)? (incorporation bias)	Y	Y	Y	Y	Y	Y
8)	Was the execution of the index test described in sufficient detail to permit replication of the test? (index test execution)	Y	Y	Y	Y (only one intercostals space examined)	Y	Y
9)	Was the execution of the reference standard described in sufficient detail to permit its replication? (reference standard execution)	Y	Y	Y	Y	Y	Y
10)	Were the index test results interpreted without knowledge of the results of the reference standard? (test review bias)	Y	Y	Y	Y	Y (confirmed with direct email communication)	Y
11)	Were the reference standard results interpreted without knowledge of the results of the index test? (reference standard review bias)	Y	Y	Y	Y	Y	Y
12)	Were the same clinical data available when test results were interpreted as would be available when the test is used in practice? (clinical review bias)	Y	Y	Y	Y	Y	Y
13)	Were uninterpretable/ intermediate test results reported? (uninterpretable test results)	U	Y	Y	Y	Y	Y
14)	Were withdrawals from the study explained? (withdrawals)	Y	Y	Y	Y	U	Y

	QUADAS Items	Brook[17]	Soldati[18]	Soldati[19]	Zhang[20]	Chung[21]	Kirkpatrick[22]	Rowan[23]
1)	Was the spectrum of patients representative of the patients who will receive the test in practice? (spectrum composition)	Y	Y	Y	Y	Y	Y	U
2)	Were selection criteria clearly described? (selection criteria)	Y	Y	Y	Y	Y	Y	Y
3)	*Is the reference standard likely to correctly classify the target condition? (reference standard)*	Y	Y	Y	Y	Y	Y	Y
4)	Is the time period between reference standard and index test short enough to be reasonably sure that the target condition did not change between the two tests? (disease progression bias)	Y	Y	Y	Y	Y	Y	Y
5)	Did the whole sample or a random selection of the sample, receive verification using a reference standard of diagnosis? (partial verification)	Y	Y	Y	Y	Y	Y	Y
6)	Did patients receive the same reference standard regardless of the index test result? (differential verification)	Y	Y	Y	Y	Y	N We included only pt with CT scans	Y
7)	Was the reference standard independent of the index test (i.e. the index test did not form part of the reference standard)? (incorporation bias)	Y	Y	Y	Y	Y	Y	Y
8)	Was the execution of the index test described in sufficient detail to permit replication of the test? (index test execution)	Y	Y	Y	Y	Y	Y	Y
9)	Was the execution of the reference standard described in sufficient detail to permit its replication? (reference standard execution)	Y	Y	Y	Y	Y	Y	Y
10)	Were the index test results interpreted without knowledge of the results of the reference standard? (test review bias)	Y	Y	Y	Y	Y	Y	Y
11)	Were the reference standard results interpreted without knowledge of the results of the index test? (reference standard review bias)	Y	Y	Y	Y	Y	U	Y
12)	Were the same clinical data available when test results were interpreted as would be available when the test is used in practice? (clinical review bias)	Y	Y	Y	Y	Y	Y	Y
13)	Were uninterpretable/ intermediate test results reported? (uninterpretable test results)	Y	Y	Y	Y	Y	Y	Y
14)	Were withdrawals from the study explained? (withdrawals)	Y	U	Y	Y	Y	Y	Y

Yes=Y, No=N, Unclear=U.
